# Heart rate variability biofeedback to reduce anxiety in autism spectrum disorder – a mini review

**DOI:** 10.3389/fpsyt.2024.1409173

**Published:** 2024-06-13

**Authors:** Helen L. Coulter, Mark P. Donnelly, Anita Yakkundi, Helen McAneney, Owen G. Barr, W. George Kernohan

**Affiliations:** ^1^ Doctoral College, Ulster University, Belfast, United Kingdom; ^2^ Institute of Nursing and Health Research, Faculty of Life and Health Sciences, Ulster University, Belfast, United Kingdom; ^3^ School of Computing, Faculty of Computing, Engineering and the Built Environment, Ulster University, Belfast, United Kingdom; ^4^ School of Health Sciences, Faculty of Life and Health Sciences, Ulster University, Belfast, United Kingdom; ^5^ Northern Ireland Public Health Research Network, Belfast, United Kingdom; ^6^ School of Medicine, Faculty of Life and Health Sciences, University of Ulster, Belfast, United Kingdom; ^7^ School of Nursing and Paramedic Science, Faculty of Life and Health Sciences, Ulster University, Londonderry, United Kingdom

**Keywords:** heart rate variability biofeedback, anxiety, autism spectrum disorder, digital health, intervention

## Abstract

There is a reported high prevalence of anxiety in people with autism spectrum disorder. This mini review appraises existing research investigating heart rate variability biofeedback to help manage symptoms of anxiety in people with autism spectrum disorder. A thorough search of electronic databases was conducted to find relevant literature. Consultation with experts and a librarian helped develop search terms following the PICO framework. Five databases were searched, and screening was undertaken using Covidence software, with the process outlined in a PRISMA flowchart. The latest review showed positive short-term effects but there is a need for long-term follow-up. Future investigations should consider device type, training settings, and control interventions. Accurate heart rate variability assessment independent of biofeedback devices is crucial. Additional measures like cortisol assessment and user feedback are recommended for comprehensive evaluation. The findings highlight progress in the evidence base and offer insight to future directions.

## Introduction

Heart rate variability (HRV) is a term used to describe the natural variability in heart rhythm which reflects the activity of both the sympathetic and parasympathetic actions of the autonomic nervous system (1). HRV is a complex variable which constantly changes according to the individual’s responses to their environment, and which also declines with age (2, 3). A detailed review of HRV metrics has been produced (4) and normative values have been reported (5, 6). A number of theoretical models have been proposed describing the links between HRV and health, mediated via connections between the heart and the brain (7–9). HRV is now frequently used as a physiological marker and is considered a sensitive indicator of the stress response (10) and an index of an individual’s ability to self-regulate behaviour (11).

Biofeedback involves monitoring physiology by actively involving the user, enabling them to learn to change their unique physiological responses to improve health (12). Several systematic reviews have been conducted highlighting the potential of biofeedback as a cost-effective digital health intervention to help people manage anxiety (13, 14). A review of the types of biofeedback modalities and devices being trialled for stress management has been carried out by Yu et al. (15). HRV measurement has been used in conjunction with sensor technology to develop a form of biofeedback now referred to as heart rate variability biofeedback or HRVB (16). HRVB involves breathing training to develop a phenomenon called Respiratory Sinus Arrhythmia (RSA), where heart rate acceleration and deceleration synchronizes with respiration and typically occurs when breathing is slowed to a rate between 4.5–7 breaths per minute (17). A guide to the process of HRVB training to develop what has been termed ‘Resonance Frequency’ breathing has been outlined (18) and the possible mechanisms of effect underlying HRVB have been described (19, 20). Several meta-analytic reviews have now demonstrated efficacy for HRVB to reduce anxiety in a range of populations (14, 21).

People with autism spectrum disorder (ASD) frequently experience high levels of anxiety (22, 23) and reviews have indicated higher prevalence rates of anxiety in young people with ASD, in comparison with typically developing peers (24). A range of interventions have been employed to treat anxiety in people with ASD (25). Despite widespread use of medication, the evidence for its effectiveness is limited and side effects and adverse events can occur (26). There is evidence for effectiveness of interventions such as cognitive behavioural therapy adapted for people with ASD (27), however the availability of interventions for anxiety is limited by difficulties with adoption of interventions (28) and lack of support and training for those working with people with ASD (29).

As a non-invasive digital health solution, HRVB may represent a useful method of engaging people with ASD. HRVB removes the complex social and communication demands of traditional cognitive and behavioural therapies (30), bypasses the risks of medication and through often intuitive digital displays, leverages the characteristic visual strengths and interests of people with ASD. People with ASD do, however, present with a wide range of differences in physiological reactions compared to neurotypical peers (31–33) and further investigation into interventions to help improve autonomic system regulation may be particularly important.

This paper presents a review conducted to assess and summarise literature that currently exists on the use of HRVB in people with Autistic Spectrum Disorder (ASD).

## Methodology

A comprehensive search of electronic databases followed by the screening of the articles was undertaken to identify relevant literature. In consultation with experts in the areas of ASD and HRV, and then further refinement with advice from a subject librarian, the search terms were developed and followed the PICO framework (Population, Intervention, Comparator and Outcome) (34). These search terms are listed in **Table 1** and were combined using Boolean logic. Five databases, CINAHL ultimate, Embase (via OVID), Medline (via OVID), PsycINFO (via OVID) and Scopus were searched to capture relevant literature across the domains represented by these databases. An initial search was carried out on 19^th^ July 2017, with no date restrictions applied, and further updated with the final search performed on 16^th^ November 2023, in which relevant publications between 2017 and 2023 were then added to form the full list of included studies in this mini review. Inclusion and exclusion criteria applied during the screening process are also outlined in **Table 1**. That is, the inclusion of peer reviewed research articles, with no restriction on date published or language. Papers were excluded if not ASD/autism, not HRV/biofeedback, no English abstract, or if it was a review article or of a single case study design. Only peer reviewed articles were included in the review. Single case studies were also excluded as they were viewed to lack generalisability due to their focus on a singular instance, limiting the applicability and reliability of findings in broader contexts.

**Table 1:** Search terms used, and inclusion and exclusion criteria applied during the screening process.

Population	Intervention	Comparator	Outcome
Autism* Asperger* Autis* ASD Autistic Spectrum Disorder Pervasive Developmental Disorder* PDD	Biofeedback	–	Heart rate variability HRV

Databases searched	Search date	Inclusion criteria	Exclusion criteria
CINAHL ultimate Embase (via OVID) Medline (via OVID) PsycINFO (via OVID) Scopus	19^th^ July 2017 and updated 16^th^ November 2023	All languages All dates Research article Peer reviewed	No English abstract Not ASD/autism Not HRV Biofeedback Not peer reviewed Review article Single case design study

In database searches an asterisk (*) denotes a wildcard and so can represent any character.

The screening process was facilitated using the software Covidence (35), which automatically removes duplicates. Articles were initially screened by title and abstract, carried out by HC & AY, followed by full text screening. A PRISMA flowchart provides an overview of this screening process (36).

Summary details of included articles were then extracted, including details of population, intervention, comparator, and outcome aspects, followed by an assessment of the study, using the CASP framework (37) as a guide, which was carried out in duplicate.

## Results

**Figure 1:**
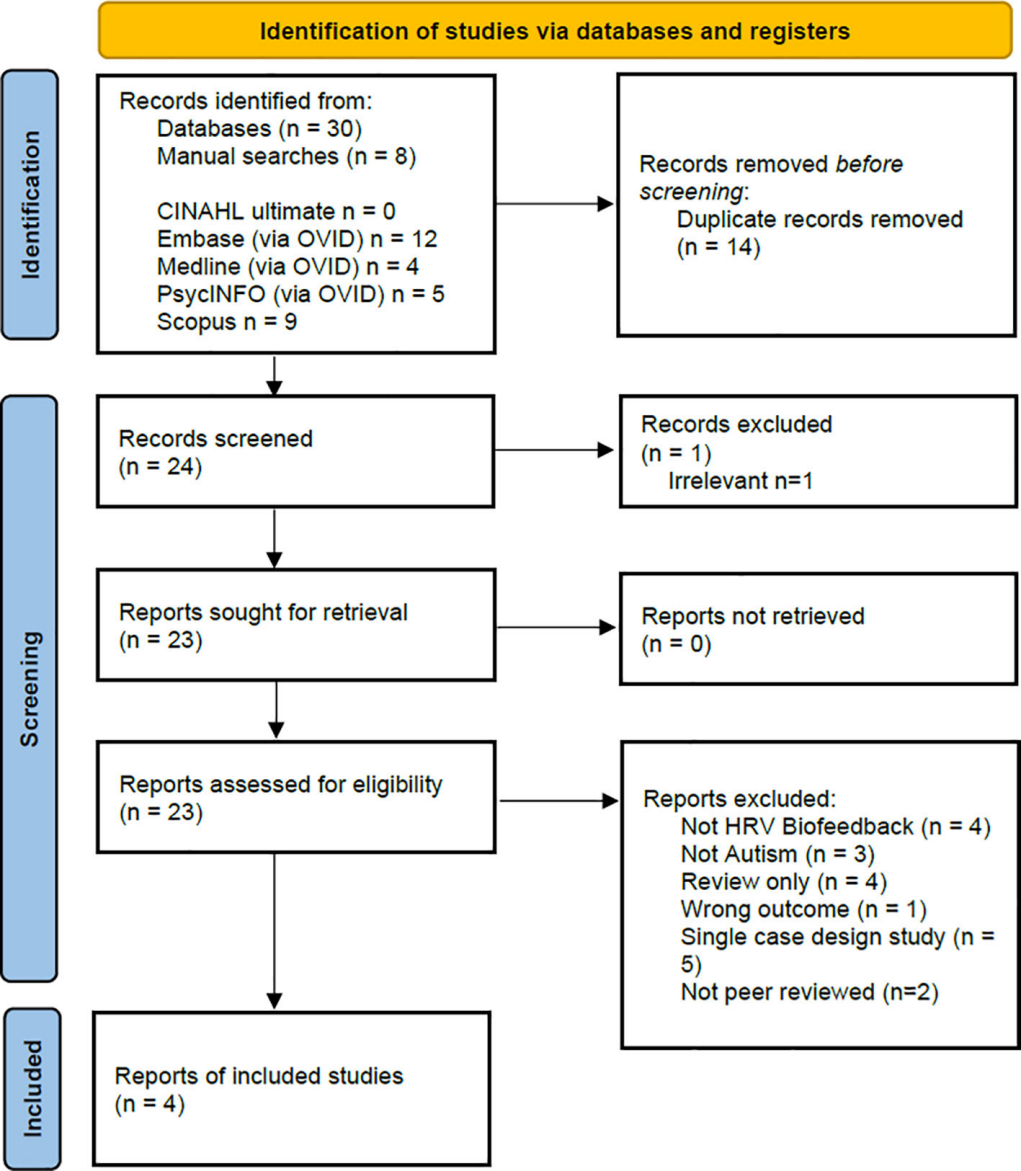
PRISMA Flowchart (36) showing the overview of the identification of and screening of studies for inclusion in this review.

An overview of the screening process is provided in the PRISMA flowchart (36) presented in **Figure 1**. In total 38 articles were returned, 30 from the searches of the five databases (CINAHL ultimate n = 0, Embase (via OVID) n = 12, Medline (via OVID) n = 4, PsycINFO (via OVID) n = 5, Scopus n = 9) and 8 additional articles from manual searches. After duplicates were removed, 24 articles were screened for inclusion with 20 articles excluded for the reasons as listed in **Figure 1**. Four remaining articles are included in this review (38–41).

**Table 2** summarises the extracted data and critical assessment of the four included articles. Two of the four articles are published within the last two years (40, 41), with two studies conducted in the USA (38, 39), one in the UK (40) and one in Belgium (41). The design of the interventions in these studies varies but all comprise exploratory or pilot studies, with appropriately small sample sizes. Across the papers, there is variation in the measures used and demographics reported.

**Table 2:** Summary of four included studies on HRVB interventions in people with ASD showing key features in design, measures, and intervention.

PICOTS framework	Papers included in review (author & year)			
	(38) McCoy (2014)	(39) Goodman (2018)	(40) Coulter (2022)	(41) Thoen (2024)
Country	USA	USA	UK	Belgium
Population				
Population	ASD & non-ASD Diagnosis method not specified	Clinical diagnosis of ASD	Clinical diagnosis of ASD + previous attendance in health services for anxiety	Clinical diagnosis of ASD
Age range	Young adults - range not specified	9–18 years	13–22 years	13–18 years
Sample size (ASD)	n=10	n=15	n=20	n=44 (Supervised HRVB 24; Sham control 20)
Demographics	Gender	Age, gender, IQ	Age, gender, sleep, medication	Formal diagnosis of ASD (excluded people with intellectual disabilities), gender, age
Intervention				
Design	2 group design	2 group design investigating two types of biofeedback intervention	2 group design investigating adoption & usability in home setting	A single-blind, randomized sham-controlled pilot trial with between subject design was used. Trial of HRVB vs Sham control over five-week period (T1), followed a second five-week study (T2) of randomized home-non supervised vs non-treatment group.
Recruitment	Autism advisor	Autism group, school, online	Therapists, flyers at clinics	Autism centre, autism research group, special education schools, clinical practices, advocacy organisations
Random allocation	No	Yes	Yes	Yes
Concealment	No	No	No	Yes
Measures				
Parent/carer questionnaire	No	Spence Anxiety Scale; Emotion Regulation Checklist; Social Responsiveness Scale; Autism Treatment Evaluation Checklist	Social Communication Questionnaire (ASD rating) Pre-post intervention report	Parents completed: Social responsiveness scale; Repetitive Behaviour Scale – Revised; Strengths and Difficulties Questionnaire
Participant questionnaire	No	No	Beck Anxiety & Beck Depression inventory/ Beck Youth Inventory Daily reports - use of device & level of stress	Adolescents self-report tools: Strengths and Difficulties Questionnaire; Perceived Stress Scale; Depression, Anxiety and Stress scale – 21; Visual Analogue Scale for sensory hypersensitivity; Visual Analogue Scale for perceived stress
Physiological testing	No	QEEG/Respiration/ECG	ECG (Actiwave Cardio)	Three lead ECG/Breathing frequency (using an elastic band with stretch sensitive sensors)/Salivary Cortisol
Adoption	Attrition rates calculated	Amount of home practice	Amount of home practice	Compliance rates for supervised HRVB and sham training (T0, T1) and home-based HRVB (T1 and T2)
Usability	No	No	System Usability Scale Debriefing report on problems/benefits	
Equipment (Manufacturer)	emWave desktop (HeartMath)	BioGraph Infiniti software 6.0 (Thought Technology)	StressEraser/Inner Balance (Helicor*) (HeartMath)	NeXus- 10 MKII and BioTrace+ software (MindMedia)
Comparator				
	Non-ASD students (n=37)	HRVB + Mu Rhythm Synchrony neurofeedback combined	Delayed intervention	Sham HRVB control
Outcome				
Analysis type	Exploratory data analysis	Statistical analysis	Pre-post analysis of mean differences in reported anxiety and depression.	Statistical analysis, including mixed effect analysis. Analysis of mean differences across HRVB and Sham groups
Findings	ASD participants showed an increase in mean HRV scores compared to students without autism in the second five weeks of the study. ASD participants showed higher median scores and less variability in their scores compared to students without ASD.	HRVB group - Increased emotion regulation and social behaviour. MRS-NFB - improvement in emotional lability, ASD behaviours, some HRV No change in Anxiety either group Results on mu suppression training contrary to some previous findings. Home practice linked to change in HRV over time.	Reduction in Anxiety post intervention No change in Depression Heart Rate correlated with level of ASD symptoms. Problems – StressEraser sensor Benefits – ‘device helped.’ Usability ratings – good	Supervised HRVB resulted in a late increase in cardiac vagal modulation in adolescents with autism. Increase in heart rate. Decrease in cortisol levels indicative of decreased stress levels immediately post HRVB, but not sustained in 5 week follow up. No significant change in psychosocial functioning and self-reported stress Authors acknowledge the confounding effects of sex, comorbidities, psychotropic medication, and severity in autism. Problems: The home-based non-supervised HRVB training was accompanied with significant decrease in compliance rate.
Time frame				
	Initial training 10 min daily x 10 session	Initial training x 4 sessions 12hrs training for each participant	Initial training 2 x30 min 5–10 min daily x 12 weeks	Phase 1: 30 min training and supervised practice session in clinic one day a week, and parallel 20min/day home session for 5 weeks. Phase 2: non-supervised 20 min home practice for 5 weeks
Setting				
	Institute Higher Education	Clinic intervention + home practice	Home intervention + practice	Clinic intervention + home practice

Assessment of studies				
Have Ethical issues been considered?	Yes	Yes	Yes	Yes
Was the Data analysis sufficiently rigorous?	No Independent physiological measurements of HRV, other than biofeedback device. No questionnaire measures of anxiety, or independent assessment of ASD symptoms were undertaken	Yes This study presents clear detailed findings in addition to a discussion regarding potential difficulties which may have influenced results	unsure As a pilot study, sample size is small, and not powered for statistical testing of intervention versus control groups. Instead, pre-post-intervention outcomes analysed on all participants. Mean and SD reported. Use of two different anxiety scales on adults and children further limited statistical testing and prevented comparison of anxiety questionnaires and HRV scores. Problems with standardizing ECG assessment across different settings.	Yes Study presents clear and rigorous methods and data analysis. Independent sample tests were used to examine differences in sociodemographic, psychosocial functioning and self-perceived stress between the groups Baseline data was collected for physiological and cortisol levels. Pre and post analysis was carried out for both phases of the study. Multiple outcome measures collected from participants as well as parents is one of the strengths of the study. Limitation: The small study sample, proper to a pilot study.
Is there a clear statement of Findings?	Rationale given for use of EDA; further rationale warranted to justify comparison between first five weeks and second five weeks of study	Yes	Yes	Yes
How Valuable is this research?	This research highlights some of the issues assessing students with ASD in a college environment. The study highlights the need to balance confidentiality with need for measures to adequately assess effectiveness of HRVB.	This research is valuable in that this is the first report of use of HRVB to reduce symptoms of ASD. The authors suggest that HRVB either alone or combined with MRS-NFB may improve features of ASD.	This research is valuable as it investigates the use of HRVB devices within the home setting.	This research is valuable because this is the first randomized control pilot study in the HRVB area of work assessing the vagal-cardiac modulation. Inclusion of physiological factors and cortisol measurements further adds to the research value.

## Discussion

Based on a review of existing literature on the use of HRVB in people with ASD, several themes emerge regarding current studies and several recommendations are made for the development of future research in this area.

Analysis of the studies reviewed here highlight wide variations in study design and the methodologies employed, which have employed different devices, training protocols and diverse outcome measurements. The current studies all provide important information on specific areas of focus and are typical of early-stage research under development however the heterogeneity of design makes direct comparisons difficult. This problem has been highlighted in several biofeedback reviews (15, 42) and there is a clear need for larger randomised control studies particularly with this new population. The most recent study reviewed in this paper, Thoen et al. (41), did employ this type of design and showed some positive effects for HRVB but also highlighted the need for more follow up to assess the longer-term effects of HRVB for ASD populations.

The type of biofeedback device used, and the type and location of training employed may also be important to consider in future investigations. Two studies reviewed here employed more real-world training in either school or home environments (38, 40) and did not adopt protocols using multi sensor devices in a clinical environment. Whilst this type of study remains problematic in terms of standardising environment conditions the need for follow up testing of devices in real world situations with clinical populations has been emphasized (43).

Resonance frequency breathing rates within individuals may not remain stable over time (44, 45) and assessing any HRVB intervention in conjunction with repeated HRV measures and age-related HRV norms is vital. The type of control intervention used may also be important. As noted by Goodman et al. (39), it is possible that simply teaching diaphragmatic breathing alone may be enough to create changes in symptoms such as anxiety. This type of slow breathing intervention has been shown to have positive effects (46, 47) and may be useful as an active control intervention in future studies to assess its effects on HRV in people with ASD.

A further issue highlighted by this review is the need to accurately assess and record HRV independently from biofeedback devices. We argue that studies should include measures of HRV measured via 12-lead ECG which are independent from the biofeedback device itself. Heart rate variability is now a commonly collected data variable which can be measured via apps on iPhones and activity trackers. However, this belies the complexity underlying the multiple influences which contribute to HRV on a moment-to-moment basis (3). The increasing number of studies now assessing HRV and the variability of data collection, analysis, and reporting, highlight the need to adhere to standardize data collection according to agreed guidelines (48). Separate independent measurement of HRV pre and post intervention is needed to elucidate whether HRVB practice does change HRV responses.

The importance of using additional measures of participants reactions is recommended to help elucidate the links between biofeedback and anxiety in ASD. The use of physiological assessments such as cortisol assessment (41) and the use of tests of EEG functioning (39) represent valuable methods of gathering vital information on physiological and neurological reactions.

The involvement of both users and carers in providing direct reports on symptoms and perceived stress in this population is also seen as a vital area which is needed. The use of participant reports on levels of stress and sources of stress (40, 41) and usability reports on device function highlight how user input can help to develop future work and highlight risks and benefits of an intervention.

Assessing the longer-term effectiveness of any intervention is important, and the use of remote monitoring to assess home practice and stress levels will be important in future work to assess adoption and usage of any intervention in this area. In addition, capturing and recording data from the biofeedback devices or from home practice reports as used by Goodman et al. (39) may help assess whether there is a dose response relationship for this type of intervention.

Several key issues are apparent regarding the specific vulnerabilities of people with ASD. Foremost in this area is the need for larger studies to establish normative values for HRV in people with ASD under a range of conditions. Several previous studies assessing the physiological responses of people with ASD have indicated that this population may have different responses to stress compared to neurotypical peers (33) and increased levels of anxiety and depression (23, 24). Assessment of autonomic nervous system functioning, and physical and mental health conditions is particularly important for people with ASD considering the increased level of these co-morbidities in this population (49–51).

In addition, the use of the ‘stress test’ paradigm may not be appropriate or indeed ethical to use for people with ASD. One feasibility study reviewed here noted that the level of ASD symptoms was correlated with increased heart rate during a stress test and noted several previously undetected mental health and cardiac health difficulties in participants during assessments (40). Recent systematic reviews have highlighted links between heart disease and ASD (52, 53) and the implications of these issues should be considered in future studies. Further work in this area should consider not employing a stress test paradigm for people with ASD and instead should consider longer term HRV recording (54) or repeated daily recordings under rest conditions and follow standardised recording and assessment guidelines (48). In addition, recording severity of symptoms in individuals with a diagnosis of ASD, as well as levels of mental and physical health symptoms pre and post intervention are important to provide more accurate information on the links between ASD and HRV.

## Conclusion

ASD is now a common condition (55) with significant economic health care costs (56, 57). Providing interventions for symptoms that affect the day to day lives of people with ASD such as anxiety have been emphasized as a vital area for research (58, 59). Despite concerns regarding overuse, there has also been a recognition of the potential for digital technology to manage the growing levels of mental health in the general population to help reduce the increasing burden of mental illness (60, 61). HRVB has been found to be an important adjunct to existing interventions for neurotypical populations with mental health conditions (62, 63) and people with ASD should not be excluded from any future research developments in this area (64, 65).

We argue that there exists evidence from recent studies to suggest the potential for this intervention to help people with ASD manage anxiety. Future studies should aim to address some of the issues outlined in this review to determine both the type and level of intervention appropriate in this vulnerable population and to further assess the mechanism of effect of HRVB.

## Author contributions

HC: Writing – original draft, Writing – review & editing. MD: Writing – original draft, Writing – review & editing. AY: Writing – original draft, Writing – review & editing. HM: Writing – review & editing. OB: Writing – review & editing. WK: Writing – original draft, Writing – review & editing.
